# An interview with Sylvia Frazier-Bowers

**DOI:** 10.1590/2176-9451.20.2.022-028.int

**Published:** 2015

**Figure:**
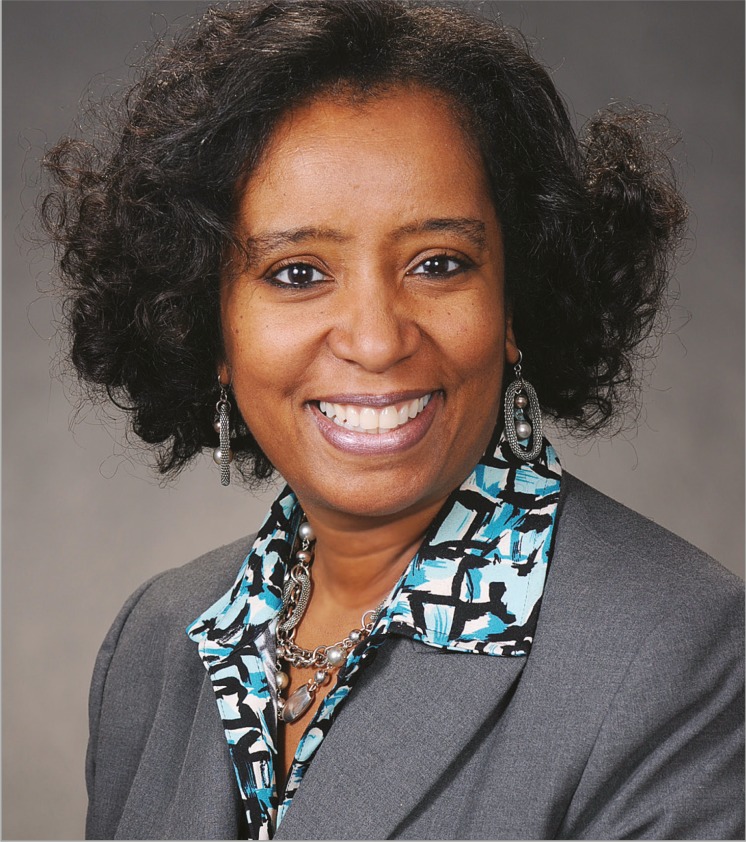

Dr. Frazier-Bowers is an associate professor at the University of North Carolina, Chapel Hill (UNC-CH), in the Department of Orthodontics. She received a BA from the University of Illinois, Urbana-Champaign, and a DDS from the University of Illinois, Chicago. After completing the NIH Dentist-Scientist Program at UNC-CH in Orthodontics (Certificate, 97') and Genetics and Molecular Biology (PhD, 99'), she completed a post-doctoral fellowship at the University of Texas Health Science Center, Houston (UTHSC), in the Department of Orthodontics. Leadership positions include president of local NC-AADR (North Carolina (2005-2006); director of the AADR Craniofacial Biology group (CBG) 2004-2007; IADR/AADR councilor for NC-AADR (2007, 2008, 2012) and for the CBG (2012-2015); member of Southern Association of Orthodontists Scientific Affairs Committee (2005-2013) and the American Association of Orthodontists Council on Scientific Affairs (2014 - Present). Dr. Frazier-Bowers also serves various editorial boards including the Journal of Dental Research and the Scientific Advisory board for the Consortium on Orthodontic Advances in Science and Technology. Her current role as faculty at UNC-CH includes conducting human genetic studies to determine the etiology of inherited tooth disorders, mentoring students at all levels, teaching graduate and pre-doctoral level Growth and Development courses and treating patients in the UNC School of Dentistry faculty practice in Orthodontics.

Dra. Frazier-Bowers é professora associada da *University of North Carolina *em Chapel Hill (UNC-CH), Departamento de Ortodontia. Recebeu o diploma de bacharel pela *University of Illinois* em Urbana-Champaign e graduou-se em Odontologia pela *University of Illinois* em Chicago. Após concluir seus estudos pelo *NIH Dentist-Scientist Program (K16)* na UNC-CH, em Ortodontia (1997) e Genética e Biologia Molecular (1999), ela finalizou seus estudos de pós-doutorado na *University of Texas Health Science Center* em Houston (UTHSC), no Departamento de Ortodontia. Ocupou várias posições de liderança, incluindo os cargos de presidente do *North Carolina Chapter of the American Association of Dental Research* (NC-AADR) entre 2005 e 2006; diretora do *AADR Craniofacial Biology Group* (CBG) de 2004 a 2007; conselheira do *International Association for Dental Research/American Association for Dental Research* (IADR/AADR) em 2007, 2008 e 2012, e do CBG de 2012 a 2015; membro do *Southern Association of Orthodontists Scientific Affairs Committee* de 2005 a 2013 e da *American Association of Orthodontists Council on Scientific Affairs* desde 2014. Dr. Frazier-Bowers também é membro do corpo editorial de vários periódicos, incluindo o *Journal of Dental Research*, e do conselho científico do *Consortium on Orthodontic Advances in Science and Technology*. Sua atual função como membro do corpo docente da UNC-CH inclui realizar estudos sobre a genética humana para determinar a etiologia de anomalias dentárias hereditárias, orientar alunos de todos os níveis, lecionar em programas de graduação e pré-doutorado em cursos sobre Crescimento e Desenvolvimento, e tratar pacientes na clínica de Ortodontia da Faculdade de Odontologia da UNC.

## Class III is one of the most challenging malocclusions to manage. Specifically, the development of an optimal diagnosis and treatment plan is difficult. Early orthopedic interventions have been advocated for skeletal Class III patients. However, many patients that are treated successfully at an early age experience relapse during subsequent growth. The prognosis of such patients can be greatly enhanced if accurate predictors of growth pattern and ultimate growth potential are identified and clinically applied. Moreover, a complete characterization of skeletal Class III individuals and future correlation with specific genetic factors holds great promise for the orthodontic specialty. In your opinion, is it clear that there are distinct types of Class III? And how this classification may help solve these cases? (Gustavo Zanardi) 

**Figure 1 -:**
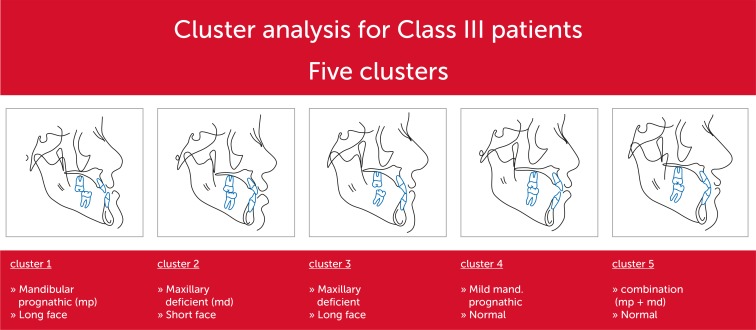
A cluster analysis of 309 individuals with Class III malocclusion revealed that five subtypes were predominant. A representative cephalometric image accompanies each subtype.

A simple answer to this question is that most orthodontists are aware that there are many subtypes of Class III malocclusion, but the agreement on what these subtypes are and how we can diagnose them is less clear. Several studies have explored the existence of different types of Class III *versus* a simplistic view of the malocclusion as originally defined by Angle. In our study^2^ we found five main subtypes that were highly relevant based on a cluster analysis of a large cohort followed by principal components analysis (Fig 1). Of the many subtypes that have been described, the two main types are maxillary deficiency and mandibular prognathism. The simple classification of these types correlates with treatment regime, that is, either you surgically move the maxilla forward (or modify growth of the maxilla) or surgically set the mandible back. The combination of these two surgical movements is also a possibility. The nuance, however, exists in the many permutations of the dentofacial relationships that can lead to a specific treatment regime. This begs the question as to whether to attempt treatment with growth modification (i.e., when and how to treat). This is due in part to a more general problem in Clinical Orthodontics; specifically that much of the diagnostic process that based on cephalometric analysis is quite controversial. To address some of these challenges in understanding, one attractive proposal would be to develop a system whereby an objective and detailed characterization of malocclusion into specific subtypes (beyond Angle's classification) could be correlated with specific haplotypes. Using Class III malocclusion as a model for this exercise, the range of the Class III phenotype should be carefully characterized first delineating, for example, between individuals with a Class III relationship, as measured by some antero-posterior (AP) determinants, such as ANB and overjet, *versus* those with a vertical component, such as downward and backward rotation of the mandible masking the AP problem. The ultimate accomplishment would be to determine the growth potential of each of these subtypes. 

## Dr. Frazier-Bowers, you are part of a select group of researchers who have studied the genetics of Class III malocclusion. Since there are relatively few groups studying this subject in the world, we can conclude that difficulties are immense. To what reasons do you attribute these difficulties: lack of a better characterization of samples due to the large phenotypic heterogeneity of Class III, or to limitations in laboratory technique of investigative genetics? (Ricardo Machado Cruz) 

**Figure 2 -:**
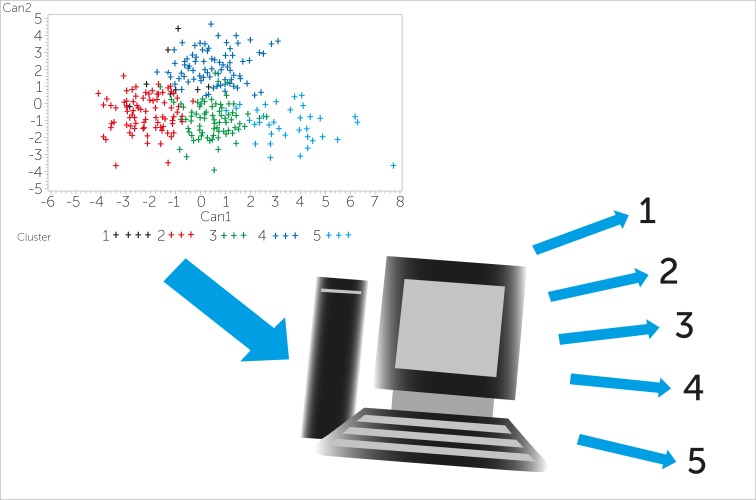
Can we use subtype classification to predict success of Class III treatment? In this schematic the clusters shown on the plot diagram are used to create a mathematical equation that can calculate the subtype of a prospective individual patient (including subtypes 1-5). A future application of these calculations may be used to predict patient success.

Actually there have been recent advances in the study of Class III malocclusion so the prospect of advancing the field of Class III treatment is very optimistic. Our understanding of growth and development of the dentofacial complex continues to evolve with the contribution of 3-D imaging and genetic advances. The difficulty; however, still lies in the fact that the Class III dentofacial phenotype is poorly understood. While studies in my laboratory have examined the Class III phenotype from the genetic and phenotypic perspective,^2^^,^ 5 we may actually lag behind in our advances in phenotypic characterization. A continuous literature review reveals that the gene discovery has progressed at a relatively impressive rate, hence, this is not where the challenge lies. Conversely, although it has been a gradual progression, the definitive characterization of the phenotypic variation remains elusive. Many studies in fact classify types as mandibular prognathic or maxillary deficient with no particular distinction of the vertical dimension. The difficulty in accomplishing the necessary phenotypic characterization is due, but not limited to two things: 1) the two dimensional tools to study dentofacial proportions is limited by its lack of depth, and 2) the availability of three-dimensional imaging is still in the nascent stages of standardization. Designing an analytic tool with the capability to provide refined and discriminatory phenotypic detail will remain a challenge in the coming years, but will be the key to maximizing our knowledge of the genetic discovery that has occurred (Fig 2).

## Would it be possible, in the near future, to create a growth prediction system based on genetic studies for individuals with Class III malocclusion? (Rhita Almeida)

It is certainly possible for this to occur in the future. We already know certain genes that are associated with Class III growth. If a comprehensive genotype phenotype correlation were completed, we could attribute certain growth patterns to certain genetic backgrounds. Accordingly, this would allow for a prediction system based on these genotype: phenotype pairs. This futuristic prediction system would require that a lot more progress be made in this area first and may realistically be a little more distant than near. A good start would be to use the information that we already have on genes that influence craniofacial growth and carefully dissect the phenotype of Class III individuals who also have genetic information available. 

## Previous studies show that Class III malocclusion presents multifactorial features with probably more than one involved gene, a significant environmental interaction and a high genetic heterogeneity, since identification of candidate loci could not be repeated in genetically distinct populations. Do you believe that chromosomal identification of the cause of Class III can be achieved in the future? And will we have the possibility of producing genetic tests that can benefit our patients? (Ricardo Machado Cruz) 

There has already been significant progress in this area. Ten loci have been associated with Class III malocclusion (mostly mandibular prognathism) and, to date, at least five genes are associated with Class III malocclusion: growth hormone receptor gene (GHR), erythrocyte membrane protein band 4.1 (EPB41), myosin 1H (Myo1H), matrillin 1 (MATN1) and dual-specificity phosphatase 6 (DUSP6). This does not immediately translate into a genetic test that will be available right away for routine use. The standard of care for genetic testing in the USA requires that it is carried out by a certified testing laboratory (i.e., with Clinical Laboratory Improvement Amendments [CLIA] certification). Currently, testing of orthodontic problems, such as Class III malocclusion or PFE, is not offered in certified laboratories. However, through research studies, such as that in my laboratory at the University of North Carolina, Chapel Hill, patients can be evaluated as part of the research protocol for certain problems. This is not meant to serve as an official test, but the results of our research evaluation of PTH1R and other candidate genes can be made available to the participant (and to the orthodontist at the request of the participant). In the future, the cost of genetic testing (i.e., of one gene) will be likely comparable to several of the other tests that orthodontists routinely call for in practice (i.e., CBCT or 3DMD). As more candidate genes are identified relating to various dentofacial characteristics, we might soon witness a change in our orthodontic diagnostic regimen. It is quite possible that in the not-so-distant future the orthodontist will collect a saliva or cheek sample for genetic tests for conditions such as PFE, root resorption or Class III malocclusion.

## Scientists are rapidly developing and employing diagnostic tests in medical diagnosis based on genomic, proteomics and metabolomics, to better predict the patients' responses to targeted therapy. This field termed "personalized medicine" combines human genome, information technology and biotechnology with nanotechnology so as to provide treatment based on individual variation versus population trends. In your opinion, how will personalized Medicine affect Dentistry and particularly orthodontic treatment? (Gustavo Zanardi) 

We are quickly approaching a time when personalized Medicine will be a part of our diagnostic regime in Dentistry as it is with Medicine.^12^ The American Society of Human Genetics (ASHG) has in fact recommended that taking a family history represents the gold standard in the diagnosis and management of medical (and by extension) dental disorders. As we enter the post-genomic era in Molecular Biology, it is the judicious combination of clinical, biological, and genetic factors that will lead to successful diagnosis and treatment of nearly all clinical disorders. Knowledge of a family history is the first step, but it is likely in the future that a saliva sample will be taken as part of the initial records routinely collected at the initial visit.

The basis for this eventual paradigm shift is that personalized Medicine and, by extension, personalized Dentistry, results from advances in translational research that aims to make connections with the genetic and molecular process involved in human disorders. As these translational studies continue to produce novel information, we will see a gradual evolution of healthcare in general, but certainly of the practice of Dentistry and Orthodontics. In fact, personalized Orthodontics has been the topic featured at meetings including the Consortium on Orthodontic Advances in Science and Technology and the upcoming College of Diplomates of the American Board of Orthodontics in 2015.

## What is the prevalence of primary failure of eruption (PFE) in the North American population? What is its prognosis? And what is the role of Orthodontics and other dental specialties in its treatment? (José Augusto M. Miguel / Rhita Almeida) 

**Figure 3 -:**
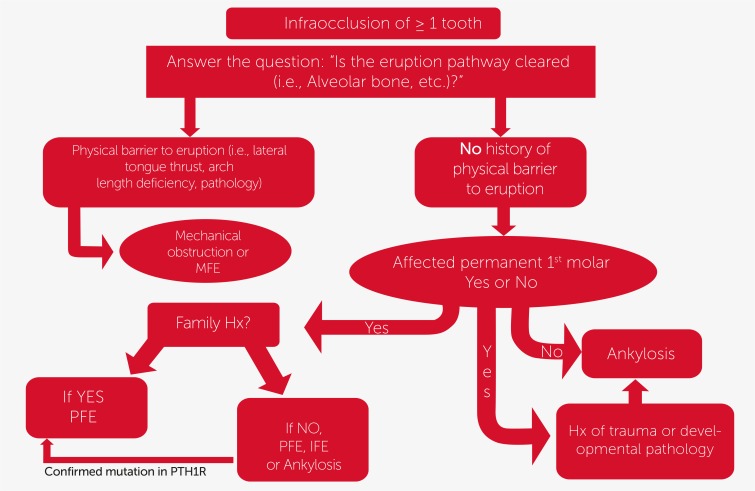
The results of our phenotype: genotype studies of a cohort with eruption disorders yielded a decision tree that serves as diagnostic criteria for eruption disorders. This rubric does not always imply a definitive treatment decision but avoids a poor treatment decision.

Primary failure of eruption is defined by a non-syndromic eruption failure of teeth in the absence of mechanical obstruction. Although descriptions of this condition have existed for more than 40 years, the exact mechanism of eruption failure, in terms of clinical and molecular parameters, is ill-defined. In recent years, there has been an increase in publications that explore the genetic etiology of PFE with most of these reports associating mutations in the parathyroid hormone receptor 1 (PTH1R) gene with PFE.^3^^,^ 6 ^,^ 7 ^,^ 10 However, very few have been truly epidemiologic in nature and therefore the actual prevalence of PFE is speculative. Several reports have estimated the occurrence of PFE to be around 1% of those who seek orthodontic treatment. Nonetheless, there have been very few studies that have accurately assessed the outcomes of PFE treatment approaches. In our studies, we determined that the best approach is to diagnose the condition in a systematic way that is definitive and evidence-based (Fig 3). While this does not solve all of our treatment mysteries, it improves the prognosis of clinical outcomes by avoiding intrusion of an entire arch that includes an affected molar tooth. It is likely that the orthodontist and possibly the pediatric dentist are those who will encounter the patient with PFE first. But the oral and maxillofacial surgeon and prosthodontist will very possibly be involved in the treatment of PFE. For instance, in cases in which the PFE condition is so severe and extraction of affected teeth and eventual drifting of teeth distal to the first molar (as in Type II PFE cases) are required, one approach may be to perform single tooth osteotomies or corticotomies to help improve the position of teeth. If the condition has a more mild manifestation, a prosthodontic approach is to treat with crowns that help camouflage the eruption problem.

## What is the differential diagnosis between primary failure of eruption, tooth ankylosis and tooth impaction? (Rhita Almeida)

We completed a study that compared PFE, mechanical failure of eruption and ankylosis, and determined that the hallmark feature of PFE is 1) at least one infraoccluded first molar; 2) a supracrestal presentation of affected teeth and, most importantly; 3) an eruption pathway that is cleared of any obstruction or alveolar bone. There is an important diagnostic distinction between isolated ankylosis, and PFE (Fig 3). If teeth distal to the more commonly unerupted first molar are normal, it might more likely be ankylosis. If they are also affected, it is likely to be PFE. If the determination is made that the diagnosis is PFE based on a familial inheritance or positive identification of a mutation in PTH1R (and likely additional genes in the not-so-distant future), then it is certain that affected teeth would be abnormal and unresponsive to orthodontic treatment. However, if it is determined that ankylosis is the correct diagnosis, the remaining teeth will be responsive to orthodontic treatment after extraction of the ankylosed tooth. 

## How can genetic analysis be associated with clinical information to improve management of primary failure of eruption? What would be the best clinical decision to treat a patient with a severe manifestation of PFE affecting several quadrants and several teeth? There is evidence of the association between PFE and osteoarthritis? (José Augusto M. Miguel / Rhita Almeida) 

The key to genetic analysis in general lies in the information that it provides about the expected biological behavior of the tissue involved. For instance, if we know that a mutation in a given gene gives rise to a specific biological reaction to orthodontic force, then we can manage that particular patient accordingly. In the case of PFE, this information will provide us with the following rubric: if a first molar is affected that with a clear bony pathway and it cannot be linked to a physical or mechanical cause, but a genetic etiology is discovered, then PFE is the likely culprit. 

PFE can occur in mild or severe forms and diagnostic distinction has been made further to include types of PFE. Previous findings in our laboratory have noted a large variability in the clinical presentation of PFE. When we evaluated a large cohort with PFE, we found that there are two distinguishable types of PFE.^4^ The first (type I) is marked by a progressive open bite from anterior to posterior of dental arches. For type I, the teeth distal to the first affected molar tooth appear to be infraoccluded to a greater extent. The second type (type II) also presents as a progressive open bite from anterior to posterior; however, there is also a more varied expression of eruption failure and greater, although inadequate, eruption of the second molar. The importance of this distinction is also the therapeutic approach that is optimal for each type. The more severe form of PFE tends to be the type I PFE that typically creates a significant posterior lateral open bite. Given the paucity of clinical studies of this malocclusion, it is not clear what the best approach is, but we know that teeth affected by PFE do not respond to orthodontic forces. There have been more anecdotal reports of osteotomies or a regional acceleratory phenomenon whereby corticotomies or microperforations are used in conjunction with orthodontic force. Other therapeutic modalities include distraction osteogenesis, which are also more rarely employed, but more importantly, the clinical outcomes have not been evaluated. The bottom line is that PFE-affected teeth do not respond to orthodontic forces alone and the determination of whether a combined surgical and orthodontic approach is highly successful simply has not been possible to make due to lack of studies in this area.

It is quite interesting to find that of those individuals affected with PFE, an association with osteoarthritis has also been observed co-segregating with PFE in some families. This does not point to a direct association between PFE and osteoarthritis, but a further exploration of this is clearly warranted. We already know that recent evidence confirms the association of osteoarthritis and a decrease in PTH1R expression in rat chondrocytes.^1^ Another study showed that treatment with an analogue of PTH decreases the progression of osteoarthritis in rats. This opens the door to a larger cohort study examining the causal relationship of PTH1R with osteoarthritis to fully test this hypothesis, since osteoarthritis otherwise occurs frequently in the population.
